# Interactive exploratory data analysis of Integrative Human Microbiome Project data using Metaviz

**DOI:** 10.12688/f1000research.24345.2

**Published:** 2021-06-21

**Authors:** Justin Wagner, Jayaram Kancherla, Domenick Braccia, James Matsumara, Victor Felix, Jonathan Crabtree, Anup Mahurkar, Héctor Corrada Bravo

**Affiliations:** 1 Department of Computer Science, University of Maryland, College Park, College Park, Maryland, 20742, USA; 2 Center for Bioinformatics and Computational Biology, University of Maryland, College Park, College Park, Maryland, 20742, USA; 3 Institute for Advanced Computer Studies, University of Maryland, College Park, College Park, Maryland, 20742, USA; 4 Institute for Genome Sciences, University of Maryland, Baltimore, Baltimore, Maryland, 21201, USA

**Keywords:** metagenomics, visualization, R/Bioconductor, Intergrative Human Microbiome Project

## Abstract

The rich data produced by the second phase of the Human Microbiome Project (iHMP) offers a unique opportunity to test hypotheses that interactions between microbial communities and a human host might impact an individual’s health or disease status. In this work we describe infrastructure that integrates Metaviz, an interactive microbiome data analysis and visualization tool, with the iHMP Data Coordination Center web portal and the *HMP2Data *R/Bioconductor package. We describe integrative statistical and visual analyses of two datasets from iHMP using Metaviz along with the *metagenomeSeq *R/Bioconductor package for statistical analysis of differential abundance analysis. These use cases demonstrate the utility of a combined approach to access and analyze data from this resource.

## Introduction

Metagenomics allows researchers to perform a microbial community census and investigate associations between host phenotype and community status. Metagenomics has been used successfully to track pathogen spread ^1^ and identify intervention strategies in childhood malnutrition ^2^. Integrative analysis of samples using multiple sequencing technologies allows for comparison at various levels of granularity. The second phase of the Human Microbiome Project (iHMP) offers a unique opportunity to test hypotheses of interactions between the microbial community and the human host. To examine the iHMP data resource, we use Metaviz ^3^, an interactive microbiome exploratory data analysis and visualization tool, and *metagenomeSeq* ^4^, an R/Bioconductor package for statistical analysis of differential abundance analysis, for combined visual and statistical analysis.

### Human Microbiome Project Phase II

The second phase of the HMP, also called the Integrative Human Microbiome Project (iHMP), consisted of focused studies of three diseases – Inflammatory Bowel Disease (IBD), Type II Diabetes (T2D), and Multi-Omic Microbiome Study: Pregnancy Initiative (MOMS-PI) ^5^. The overall goal of the project was to identify associations between human microbiome community census data and the three diseases. Each of the studies were structured for the specific disease and consisted of separate cohorts.

### Metaviz

Metaviz ^6^ is a web-based interactive visualization tool for microbiome data analysis. The architecture consists of a JavaScript front-end suite of charts (based on D3.js and Canvas) and a navigation component that lets users select portions of taxonomic hierarchies to visualize and analyze. Metaviz supports two backend data stores – a graph database and the *metavizr* R/Bioconductor package. Metaviz is tightly integrated with the *metagenomeSeq* statistical testing package so differential abundance testing results can be viewed directly in a Metaviz session. We host an instance of Metaviz that we call the UMD Metagenome Browser ( http://metaviz.cbcb.umd.edu).

### Related work

Visualization tools for large-scale sequencing consortium projects provide a mechanism to explore and interact with data from multiple studies. These applications help users analyze individual datasets and examine trends across the entire project. MAGI is a web-application that enables a user to examine data from TCGA data ^7^. The Earth Microbiome Project provides an interactive visualization web-application to analyze its data ^8^. EMPeror offers interactive 3D visualizations of PCA plots to show distances between microbiome samples ^9^. QIIME packages a number of tools for static plotting of Principal Coordinate Analysis and stacked bar plots ^10^. MetaPhlAn2 uses a visualization package called GraphPhlan to produce phylogenetic trees and other plots ^11^. The *HMP2Data* R/Bioconductor provides processed 16S sequencing data from the iHMP project in Bioconductor data structures ^12^. We implemented Metaviz using design patterns from Epiviz ^13^, an interactive epigenetics visualization tool, that visualizes data from a variety of epigenetic sequencing projects. We show how we leverage the microbiome measurement-based design of Metaviz to implement interactive exploration and hypothesis-testing of the iHMP resource.

## Implementation

### Metaviz integration with HMP infrastructure

The HMP Data Access and Coordination Center maintains a data repository and web portal ( https://www.ihmpdcc.org). From this web portal, users can browse metadata for datasets, raw sequencing files, and processed files including taxonomic community profile abundance matrices. We implemented several mechanisms to interact with the HMP data resources through Metaviz ^6^.

### Data loaded into UMD Metagenome Browser

**Figure 1.**
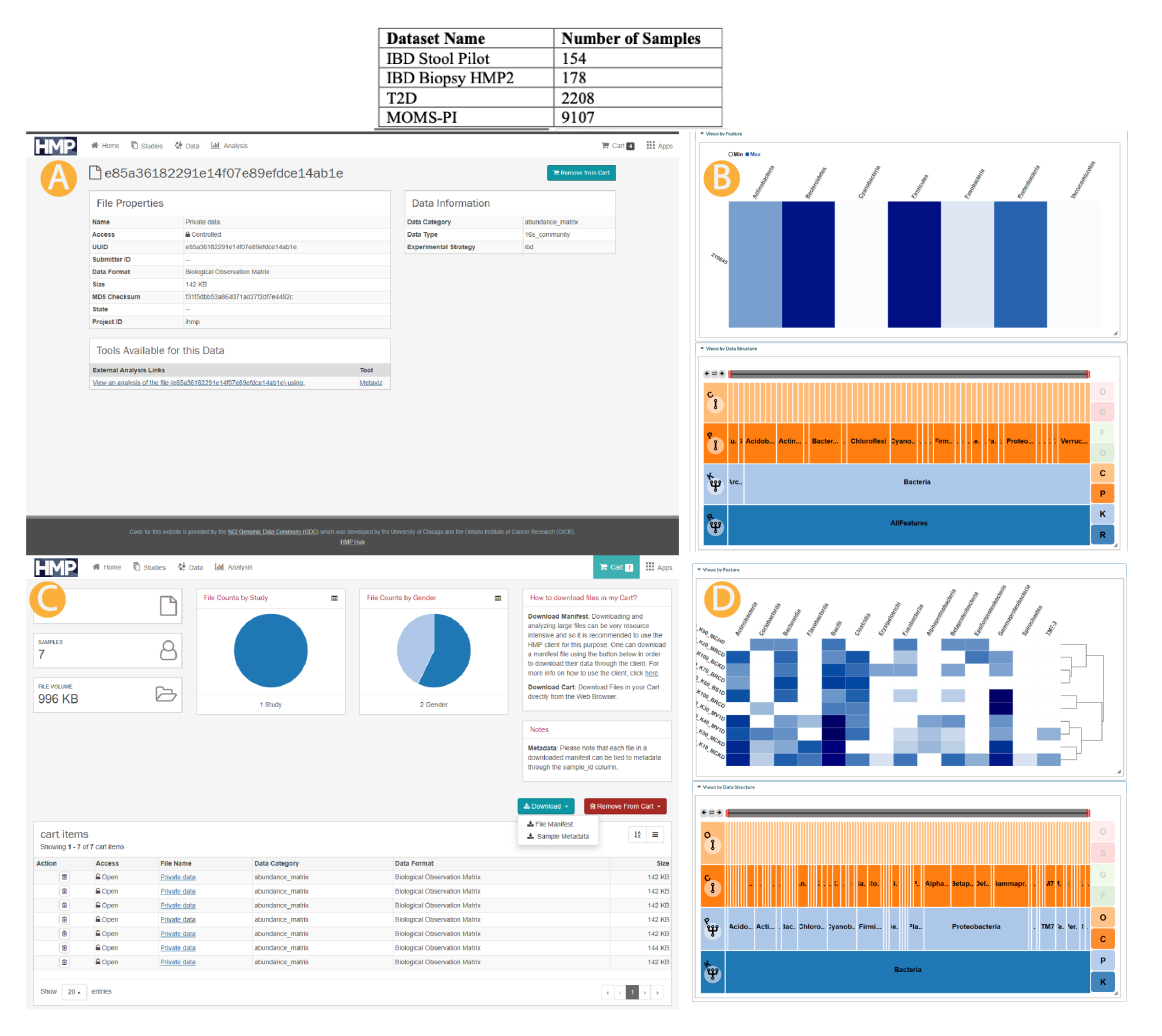
Metaviz and iHMP data infrastructure integration. Top: iHMP data accessible through the UMD Metagenome Browser. Middle ( **A**, **B**): Single sample link from data portal to UMD Metagenome Browser. Bottom ( **C**, **D**): Multiple samples manifest file upload and selection to UMD Metagenome Browser. We provide several mechanisms to access the HMP dataset from Metaviz. First, we loaded the three datasets (IBD, T2D, and MOMS-PI) into the hosted instance of Metaviz directly. A user can choose any of these datasets from the data selections screen then samples can be chosen within each dataset. We also link to the HMP Data Portal for single samples as shown in the Middle panel ( **A**, **B**). Finally, the HMP Data Portal provides a “cart” functionality where a user can select multiple samples and download a manifest listing those files ( **C**). A user can upload a manifest file containing selections from the 16S community abundance profiles from the same dataset (IBD, T2D, or MOMS-PI) to the UMD Metagenome Browser and a new Metaviz workspace is created with those files ( **D**).

We loaded the 16S community profile abundance matrices for the samples from the IBD, T2D, and MOMS-PI studies as provided by the *HMP2Data* Bioconductor package ^12^ into the UMD Metagenome Browser. A user can select each dataset from the application start screen. Figure 1 details the number of samples, with metadata to the extent available as of May 2020 from the *HMP2Data* package, from each project currently available in the UMD Metagenome Browser.

### HMP Data Portal linking to Metaviz

When browsing the samples available from the HMP Data Portal, a user can view an individual abundance matrix in Metaviz using the Metaviz tool link from the file description page. When the user clicks the link, a redirect occurs to the UMD Metagenome Browser with a new workspace containing a FacetZoom navigation utility and a heatmap for that sample. Figure 1A shows the direct link functionality for samples in the IBD dataset and resulting workspace in Metaviz ( Figure 1B).

### Metaviz import of Data Portal Manifest

In the HMP data portal, a user can select files with a shopping cart utility and download the selections as a manifest file. In the UMD Metagenome Browser, the user can upload the manifest file to create a Metaviz workspace on the fly for those samples. Currently, only files from the same project can be viewed in one workspace. Resolving taxonomic hierarchies across datasets in Metaviz is future work that could use a utility such as the *metagenomeFeatures* R/Bioconductor package ^14^. Figure 1C shows the manifest file workflow for samples from the IBD dataset and resulting workspace in Metaviz ( Figure 1D).

### Metaviz usage

For ease of use, we provide tutorials at https://epiviz.github.io/tutorials/metaviz/. As a community resource, we plan to update the Metaviz database within a month of Bioconductor releases of HMP2Data. We maintain links to the HMP Data Portal through the update of *HMP2Data* package URLs and provide default workspaces for the HMP2 datasets as well as those in *HMP16SData* R/Bioconductor package ( https://epiviz.github.io/metaviz-workspaces/). For generating data summaries, we recommend using the HMP2Data package with appropriate R libraries to summarize sample information and the interactive HMP Data Portal for data summaries over different samples or study attributes. We provide instructions in the *metavizr* vignette to handle visualizing data that would be added to an analysis session like protected variables from dbGAP.

## Operation

The HMP Data Portal and Metaviz are web applications that can run in any modern browser. We recommend using Firefox (version 65 or later) or Chrome (version 65 or later) for best performance. *Metavizr* is a Bioconductor package and general guidelines from Bioconductor for requirements and installation should be followed ( https://bioconductor.org/install/).

## Use cases

### *metavizr* analysis of WGS vs 16S data from same samples

**Figure 2.**
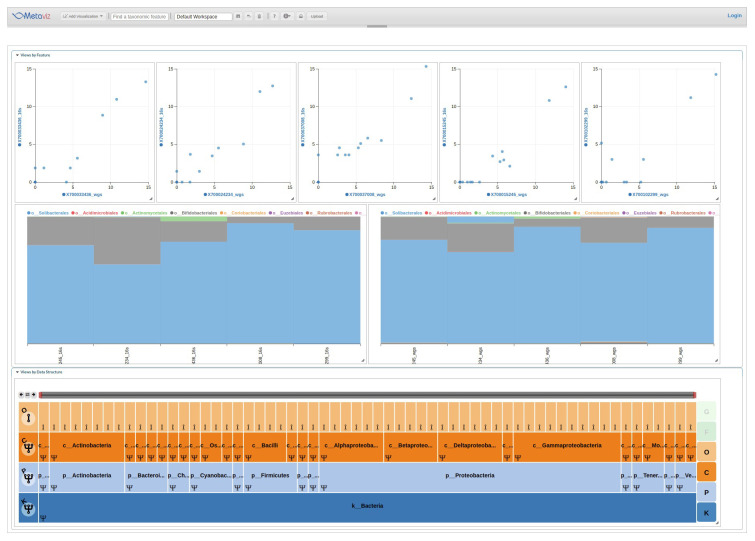
Comparison between 16S and WGS taxonomic profiling using metavizr. We identified taxa present in the taxonomic hierarchy for each method and created a merged dataset. A FacetZoom (bottom) shows the common taxonomic features, two Stacked Plots (middle) show the proportion of all features aggregated to the Order level, and a set of scatter plots (top) for samples with WGS abundance on the X-axis and 16S abundance on the Y-axis. For WGS, the relative proportion output from MetaPhlan for taxa at the order level were transformed to counts per 1000 reads. The scatter plots show the variability in taxonomic community census estimates between sequencing methods. A static similar stacked plot visualization is shown in the main HMP consortium manuscript at the genus and species level across samples for comparison ^16^. Metaviz allows users to make specific selections of the FacetZoom to compare taxa at various levels. The scatter plot also allows comparison at single sample resolution. Code to create this Metaviz session is available at the following gist: https://gist.github.com/jkanche/9216d465d18ab106be7a43f5340eb38a.

In the IBD cohort of the iHMP dataset, investigators sequenced a subset of samples using whole metagenome and 16S sequencing. We developed functions in *metavizr* to compare 16S and whole metagenome data for individual samples. Using the taxonomic profiles of the IBD samples, we matched the taxonomic features discovered with both sequencing methods. With this subset of features, we generated a single taxonomic hierarchy then loaded the 16S and whole metagenome abundance measurements into a *metavizr* object. Figure 2 shows an example analysis with stacked plots and scatter plots that link to a single FacetZoom to compare the degree of consistency of the data across sequencing methods.

### IBD dataset

The IBD study consisted of two phases: a pilot, which we refer to in this work as the IBD Stool Pilot, and a larger phase that we call IBD iHMP. We use the taxonomic profiles for each phase available from the *HMP2Data* package and use the same taxonomic classification identifiers in the package. To upload project data on to the UMD Metagenome Browser, we extracted 16S count table and taxonomic annotation using the *otu_table*() and *tax_table*() methods of *HMP2Data* package. We then use *metagenomeSeq* and *metavizr* to import the count data along with taxonomy and sample metadata into a neo4j graph database ^15^ using the *metavizr* neo4j import functionality. We used Metaviz ^6^ for exploratory analysis and *metagenomeSeq* for confirmatory statistical testing. We examined the IBD Stool Pilot and IBD iHMP dataset separately.

### IBD Stool Pilot dataset

**Figure 3.**
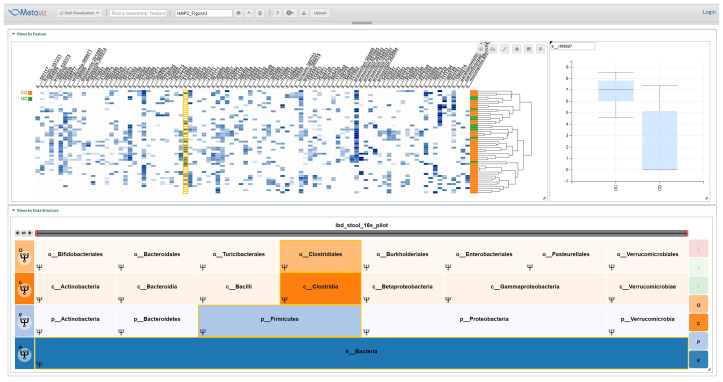
Metaviz Analysis of IBD Stool 16S Pilot Dataset. A Metaviz workspace with a FacetZoom taxonomic hierarchy, heatmap, and boxplot for the specific feature in this instance s__:369227. This identifier was from the community abundance profiles available from the HMP2Data package. We identified taxonomic features at each level of the hierarchy using this integrated view and the results for features with a potential differential abundance are listed in Supplementary Table 1. The workspace is available at: http://metaviz.cbcb.umd.edu/?ws=oLq2Fr9AwVc.

The IBD Stool Pilot dataset contains 16S and whole metagenome sequencing results of stool samples from 41 Crohn’s disease (CD) subjects and 10 ulcerative colitis (UC) subjects. We focused our analysis on 16S sequencing and used Metaviz to visually identify taxa that showed a difference in abundance between CD and UC subjects. Figure 3 shows a typical visualization.

We also used *metagenomeSeq* to test the differential abundance of features aggregated to each level of the taxonomy using the *fitFeatureModel* method that is based on a zero-inflated log-normal linear model. As shown in Table 1, two species had an absolute log fold-change greater than 1 and adjusted (Benjamini-Hochberg) p-value less than 0.1. Visually inspecting the IBD Stool Pilot data by aggregating counts to each level of the taxonomy we found the following features appearing differentially abundant: “c__Betaproteobacteria”, “o__Burkholderiales”, “f__Ruminococcaceae”, “g__Lachnospira”, “g__[Ruminococcus]”, “g__Faecalibacterium”, “s__:589277”, “s__:333166”, “s__:564806”, “s__:369227”, “s__:358104”, “s__:369486”, “s__gnavus:360015”, “s__prausnitzii:851865”. These taxonomic features describe paths in the taxonomy of the Kingdom Bacteria that was derived from the SILVA ^17^ database. The documentation ( https://ibdmdb.org/tunnel/public/HMP2/16S/1806/products) for the abundance profiles used in this analysis denotes that this taxonomic string was generated with the sequence of an OTU derived with the UPARSE algorithm ^18^ that was mapped to the SILVA database. Among these features, "c__Betaproteobacteria" refers to Class Betaproteobacteria, "o__Burkholderiales" to Order Burkholderiales, while values towards the leaves of the taxonomy refer to entries in the SILVA database that have an identifier and a sequence but have not been provided formal names in the binomial nomenclature system. Comparing the visual analysis results and the *metagenomeSeq* differential abundance testing results in Table 1 shows that the taxonomic feature s__:369227 (member of the Lachnospiraceae family which are strictly anaerobic ^19^) was identified using both methods. Members of Lachnospiraceae are abundant in human intestinal tracts and have been linked specifically to production of butyric acid ^19^. Also, colonization with a specific strain of Lachnospiraceae in obese mice has been linked to development of hyperglycemia ^20^. The second taxon, s__:363232, is a member of the genus *Dorea* which has recently been shown to be associated with diarrhea predominant irritable bowel syndrome ^21^.

**Table 1.** metagenomeSeq analysis of IBD Stool 16S Pilot dataset.

	Log fold change	se	p-value	Adjusted p-value
s__:369227	1.864583442	0.431193725	1.53061E-05	0.000734694
s__:363232	1.193035074	0.275415013	1.47914E-05	0.000734694

We used the fitFeatureModel of *metagenomeSeq* and aggregated counts to each level of the taxonomic hierarchy. Our analysis identified s__:369227 under family *Lachnospiracea* and s__:363232 under genus *Dorea* as differentially abundant between samples from subjects diagnosed with Ulcerative Colitis and Crohn’s Disease.

### IBD iHMP

**Figure 4.**
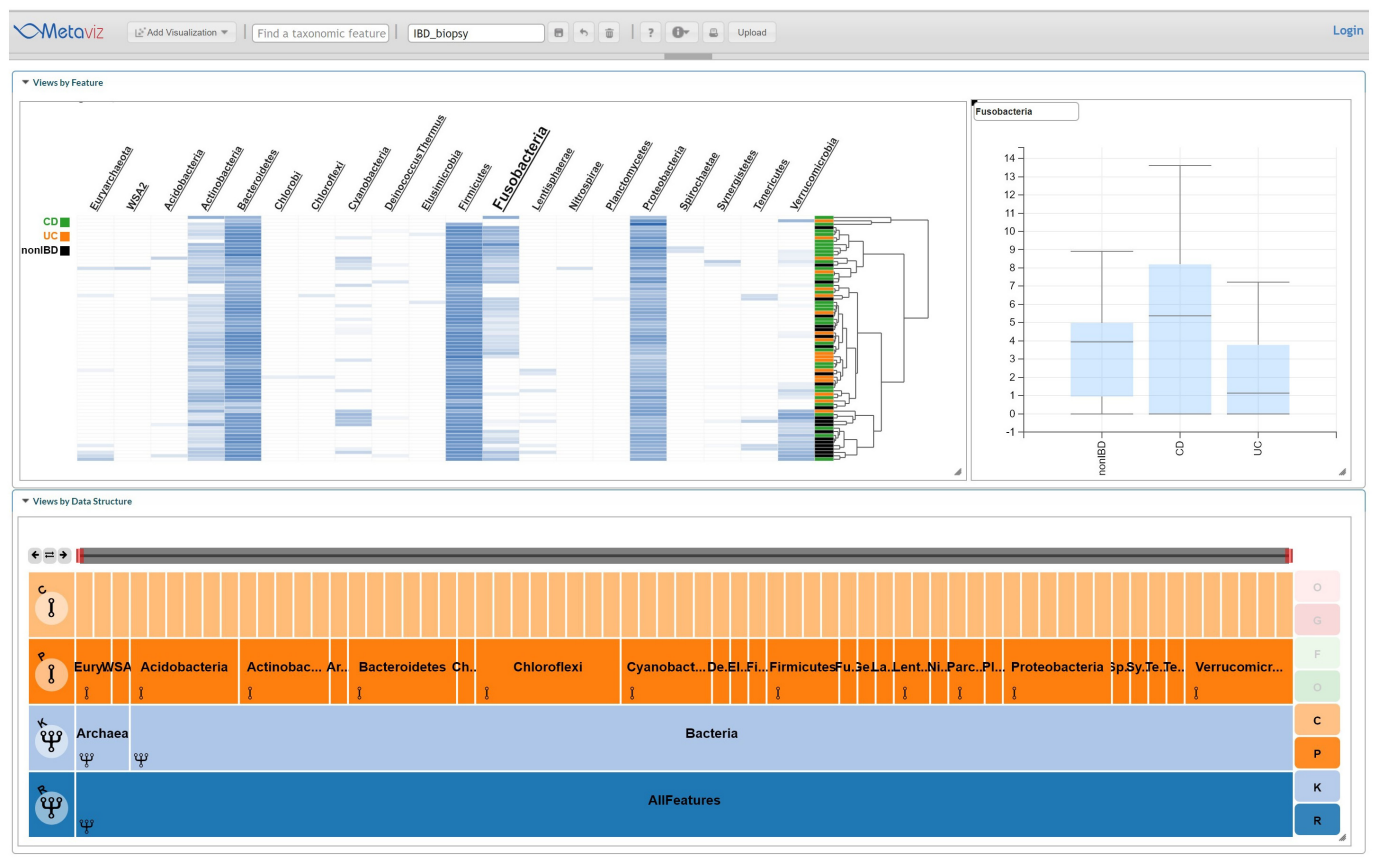
IBD Biopsy iHMP Multiple Groups Analysis. Using statistical analysis we identified taxonomic features that showed a difference in abundance between the three subject diagnosis categories: UC, CD, or nonIBD in the Fusobacteria phylum. This Metaviz workspace is available at: http://metaviz.cbcb.umd.edu/?ws=wHsHT56U8Ru.

The IBD iHMP dataset consists of samples from subjects with CD, UC, and those without IBD (nonIBD). For these samples, we analyzed the 16S sequencing data of an ileum biopsy from the first visit for each subject, which yielded 72 samples with 32 from CD, 18 from CD, and 22 from nonIBD. We used *metagenomeSeq* to compute an F-statistic to determine if any taxonomic feature is associated with at least one group using the *fitZig* method (based on a zero-inflated Normal linear model on log-transformed counts appropriate for multi-category experiment designs). Figure 4 shows an example using Metaviz to visualize abundance profiles for phylum Fusobacteria, which was found to be differentially abundant across the three groups. Differential abundance of members of this phylum has previously been reported in studies of IBD ^22^. Analysis code and results are available as *Extended data* ^23^.

## Conclusion

In this work we presented software infrastructure linking Metaviz to the iHMP data resources ^6^. We detailed the 16S taxonomic community profile data from iHMP available in the UMD Metagenome Browser. We then described linking the UMD Metagenome Browser to the iHMP Data Portal for single files and the manifest file utility for multiple file selections. We also performed visual exploratory and confirmatory differential abundance analysis of data from the IBD study. We first visualize 16S and whole metagenome sequencing abundance measurements for the same samples in *metavizr*. Then we use Metaviz and *metagenomeSeq* to analyze two datasets, IBD Stool Pilot and iHMP IBD, to examine taxonomic feature abundances in samples from UC, CD, and those without IBD. These illustrative analyses demonstrate the utility of Metaviz for integrative analysis with the HMP data resources. Visual inspection of taxonomic features coupled with statistical testing provides an effective mechanism to explore and test associations between bacterial communities and their human hosts.

## Data availability

### Source data

The 16S abundance matrices for IBD, T2D and the MOMS-PI studies were downloaded from the *HMP2Data* Bioconductor package. These datasets are then loaded into the neo4j graph database using import methods available in the *metavizr* ^24^ Bioconductor package. These import scripts are available at https://gist.github.com/jkanche/c57d8220a33b41e21c4f6769a7aef7e4.

### Extended data

Figshare: Differential Abundance Analysis - IBD ( Figure 4). https://doi.org/10.6084/m9.figshare.12404222.v2 ^23^.

This file contains differential abundance analysis code and results.

Extended data are available under the terms of the Creative Commons Attribution 4.0 International license (CC-BY 4.0).

## Software availability

**Metaviz is available at:** http://metaviz.cbcb.umd.edu.

**Source code available from:** https://github.com/epiviz/Metaviz.

**Archived source code at time of publication:** http://doi.org/10.5281/zenodo.3871869 ^6^.

**License:** Artistic License version 2.0.
