# Supplementary material for: Interactive exploratory data analysis of Integrative Human Microbiome Project data using Metaviz
Source: F1000Res. 2021 Jun 21;9:601. Originally published 2020 Jun 12. [Version 2] doi: 10.12688/f1000research.24345.2 (PMC7366035; doi:10.12688/f1000research.24345.2)
Supplement: Peer review history

# Peer review: Reviewer response for version 2

**DOI:** 10.5256/f1000research.56081.r87967

**Published:** 2021-06-23

**Authors:** Ekaterina Smirnova

**Affiliations:** 1 Department of Biostatistics, Virginia Commonwealth University, Richmond, USA

No further comments to make.

Are the conclusions about the tool and its performance adequately supported by the findings presented in the article?

Yes

Is the rationale for developing the new software tool clearly explained?

Yes

Is the description of the software tool technically sound?

Yes

Are sufficient details of the code, methods and analysis (if applicable) provided to allow replication of the software development and its use by others?

Partly

Is sufficient information provided to allow interpretation of the expected output datasets and any results generated using the tool?

Yes

Reviewer Expertise:

Biostatistics (working on Human Microbiome Project data)

I confirm that I have read this submission and believe that I have an appropriate level of expertise to confirm that it is of an acceptable scientific standard.

# Peer review: Reviewer response for version 1

**DOI:** 10.5256/f1000research.26859.r64702

**Published:** 2020-07-20

**Authors:** Levi Waldron

**Affiliations:** 1 Graduate School of Public Health and Health Policy, Institute for Implementation Science in Population Health, City University of New York, New York, NY, USA

The authors present a web tool for exploratory analysis of iHMP data, based on their existing metaviz software. The figures from the manuscript were easy to reproduce using the links and code provided. It seems like a useful new way to view, explore, and download iHMP data. I have only a couple minor comments:

1. The figures are low-resolution and text is a bit blurry. The lightest yellow text in Figure 2 isn't readable.
2. From http://metaviz.cbcb.umd.edu/ it's not immediately obvious how to find the iHMP (HMP2) data.  IBD and T2D do appear a ways down a list of available datasets, but this list takes a very long time to load for me ("loading datasets and sample annotations..."). I didn't find MOMS-PI but I may not have waited long enough. It would be worth providing direct links in the manuscript to workspaces for each dataset, like is already done for Figure 3.
3. Perhaps explain the feature variables like c__Betaproteobacteria, o__Burkholderiales, f__Ruminococcaceae, g__Lachnospira, g__[Ruminococcus], g__Faecalibacterium, s__:589277, s__:333166, s__:564806, s__:369227, s__:358104, s__:369486, s__gnavus:360015, s__prausnitzii:851865 for readers who are familiar with scientific taxonomy names but not this format.

Are the conclusions about the tool and its performance adequately supported by the findings presented in the article?

Yes

Is the rationale for developing the new software tool clearly explained?

Yes

Is the description of the software tool technically sound?

Yes

Are sufficient details of the code, methods and analysis (if applicable) provided to allow replication of the software development and its use by others?

Yes

Is sufficient information provided to allow interpretation of the expected output datasets and any results generated using the tool?

Yes

Reviewer Expertise:

bioinformatics, bioonductor, metagenomics, human microbiome analysis.

I confirm that I have read this submission and believe that I have an appropriate level of expertise to confirm that it is of an acceptable scientific standard.

# Response

**Published:** 2021-04-21

**Authors:** Hector Corrada Bravo

**Affiliations:** University of Maryland, USA

**Reviewer Comment: **The figures are low-resolution and text is a bit blurry. The lightest yellow text in Figure 2 isn't readable.

**Author Response**: We updated the color scheme for Figure 2. We also updated figures with a higher resolution.

**Reviewer Comment: **From http://metaviz.cbcb.umd.edu/ it's not immediately obvious how to find the iHMP (HMP2) data.  IBD and T2D do appear a ways down a list of available datasets, but this list takes a very long time to load for me ("loading datasets and sample annotations..."). I didn't find MOMS-PI but I may not have waited long enough. It would be worth providing direct links in the manuscript to workspaces for each dataset, like is already done for Figure 3.

**Author Response**: We developed default workspaces for IBD, T2D, and MOMS-PI as well as put them at https://epiviz.github.io/metaviz-workspaces/

**Reviewer Comment: **Perhaps explain the feature variables like c__Betaproteobacteria, o__Burkholderiales,       f__Ruminococcaceae, g__Lachnospira, g__[Ruminococcus],               g__Faecalibacterium, s__:589277, s__:333166, s__:564806, s__:369227, s__:358104, s__:369486, s__gnavus:360015, s__prausnitzii:851865 for readers who are familiar with scientific taxonomy names but not this               format.              

**Author Response**: We added the following text to the manuscript: “These taxonomic features describe paths in the taxonomy of the Kingdom Bacteria that was derived from the SILVA ^16^ database. The documentation ( https://ibdmdb.org/tunnel/public/HMP2/16S/1806/products) for the abundance profiles used in this analysis denotes that this taxonomic string was generated using the sequence of an OTU derived with the UPARSE algorithm ^17^ that was mapped to the SILVA database. Among these features, "c__Betaproteobacteria" refers to Class Betaproteobacteria, "o__Burkholderiales" to Order Burkholderiales, while values towards the leaves of the taxonomy refer to entries in the SILVA database that have an identifier and a sequence but have not been provided formal names in the binomial nomenclature system”

# Peer review: Reviewer response for version 1

**DOI:** 10.5256/f1000research.26859.r64700

**Published:** 2020-07-16

**Authors:** Edoardo Pasolli

**Affiliations:** 1 University of Naples Federico II, Naples, Italy

The manuscript by Justin Wagner *et al*. presents an infrastructure that integrates Metaviz, an interactive microbiome data analysis and visualization tool that was previously published by the same authors, with the iHMP Data Coordination Center web portal and the HMP2Data R/Bioconductor package. The authors give an overall overview of the infrastructure in addition to a couple of use cases.

 This is a nice and timely contribution that helps in using the large and complex set of available HMP data. The manuscript is already well structured, I have just few comments:

1. Please check all the links reported in the text. For example, the link to the data repository and web portal (https://ihmpdcc.org) doesn't seem to work.
2. How will the proposed infrastructure deal with likely updates (in terms of new data) of the HMP web portal?
3. Which are the options available to save/export the results generated in the proposed infrastructure? Please comment more about this in the text.

Are the conclusions about the tool and its performance adequately supported by the findings presented in the article?

Yes

Is the rationale for developing the new software tool clearly explained?

Yes

Is the description of the software tool technically sound?

Yes

Are sufficient details of the code, methods and analysis (if applicable) provided to allow replication of the software development and its use by others?

Partly

Is sufficient information provided to allow interpretation of the expected output datasets and any results generated using the tool?

Yes

Reviewer Expertise:

Microbiome and bioinformatics

I confirm that I have read this submission and believe that I have an appropriate level of expertise to confirm that it is of an acceptable scientific standard.

# Response

**Published:** 2021-04-21

**Authors:** Hector Corrada Bravo

**Affiliations:** University of Maryland, USA

**Reviewer Comment: **Please check all the links reported in the text. For example, the link to the data repository and web portal ( https://ihmpdcc.org) doesn't seem to work.                 

**Author Response**: We have updated the link to http://www.ihmpdcc.org and checked that it resolves.

**Reviewer Comment: **How will the proposed infrastructure deal with likely updates (in terms of new data) of the HMP web portal?   

**Author Response**: We updated the manuscript with the following: “As a community resource, we plan to update the Metaviz database within a month of Bioconductor releases of HMP2Data. We maintain links to the HMP Data Portal through the update of HMP2Data package URLs and provide default workspaces for the HMP2 datasets as well as those in HMP16SData R/Bioconductor package ( https://epiviz.github.io/metaviz-workspaces/)”

**Reviewer Comment: **Which are the options available to save/export the results generated in the proposed infrastructure? Please comment more about this in the text.                   

**Author Response**: We added the following tutorial to https://epiviz.github.io/tutorials/metaviz/save_plots/

# Peer review: Reviewer response for version 1

**DOI:** 10.5256/f1000research.26859.r64704

**Published:** 2020-07-06

**Authors:** Ekaterina Smirnova

**Affiliations:** 1 Department of Biostatistics, Virginia Commonwealth University, Richmond, USA

Manuscript “Interactive exploratory data analysis of Integrative Human Microbiome Project data using Metaviz” by Wagner *et al. *provides a novel interactive tool for visualization of interactive Human Microbiome Project (iHMP) Data. This tool encompasses both the HMP Data coordination center (DAC) portal as well as the Bioconductor HMP2Data package. Given the size and complexity of HMP data, this visualization tool allows users to explore major data summaries and patterns. As such, this work is very timely and is highly recommended for publication.

 Major comments:

1. How does the tool handle HMP DAC and HMP2Data package data updates? There is discussion that user can upload additional data manifest from HMP DAC portal, but what if the data already available thought Metaviz tool is updated on the DAC or Bioconductor?
2. Is there an option to download data summaries? Such as demographics table, number of samples by body site, etc.
3. MOMS-PI and  T2D studies have protected meta data available through dbGap. If users apply for dbGap and get access, id there an option to merge meta data by sample ids and visualize using this tool?
4. It would be helpful to provide instructions how to create diversity boxplots by disease status (as in Figure 3). I tried creating these but could not produce them on the same plot.
5. I recommend making alpha diversity plots by disease status a default plot when the data is selected using visualization tool.

Are the conclusions about the tool and its performance adequately supported by the findings presented in the article?

Yes

Is the rationale for developing the new software tool clearly explained?

Yes

Is the description of the software tool technically sound?

Yes

Are sufficient details of the code, methods and analysis (if applicable) provided to allow replication of the software development and its use by others?

Partly

Is sufficient information provided to allow interpretation of the expected output datasets and any results generated using the tool?

Yes

Reviewer Expertise:

Biostatistics (working on Human Microbiome Project data)

I confirm that I have read this submission and believe that I have an appropriate level of expertise to confirm that it is of an acceptable scientific standard.

# Response

**Published:** 2021-04-21

**Authors:** Hector Corrada Bravo

**Affiliations:** University of Maryland, USA

**Reviewer Comment: **How does the tool handle HMP DAC and HMP2Data package data updates? There is discussion that user can upload additional data manifest from HMP DAC portal, but what if the data already available thought Metaviz tool is updated on the DAC or Bioconductor?  

        

**Author Response**: We updated the manuscript with the following: “As a community resource, we plan to update the Metaviz database within a month of Bioconductor releases of HMP2Data. We maintain links to the HMP Data Portal through the update of HMP2Data package URLs and provide default workspaces for the HMP2 datasets as well as those in HMP16SData R/Bioconductor package ( https://epiviz.github.io/metaviz-workspaces/)”

**Reviewer Comment: **Is there an option to download data summaries? Such as demographics table, number of samples by body site, etc.

**Author Response**: We updated the manuscript with the following: “For generating data summaries, we recommend using the HMP2Data package with appropriate R libraries to summarize sample information and the interactive HMP Data Portal for data summaries over different samples or study attributes. We provide instructions in the metavizr vignette to handle visualizing data that would be added to an analysis session like protected variables from dbGAP”

**Reviewer Comment: **MOMS-PI and T2D studies have protected meta data available through dbGap. If users apply for dbGap and get access, id there an option to merge meta data by sample ids and visualize using this tool?

**Author Response**: We added a vignette to the metavizR Bioconductor package ( https://github.com/epiviz/metavizr/pull/2/commits/756cc049a2c9264355e15df42cfa64d71ee141ae#diff-46f4a8e59ff35c66f1220d9cdc6abf687d92771b424f4a6dfed1434c998cd500) for added a protected variable and then generate new plots with it.

**Reviewer Comment: **It would be helpful to provide instructions how to create diversity boxplots by disease status (as in Figure 3). I tried creating these but could not produce them on the same plot.

**Author Response**: We added a tutorial at metaviz.org for including a diversity plot ( https://epiviz.github.io/tutorials/metaviz/diversity_plot/).

**Reviewer Comment: **I recommend making alpha diversity plots by disease status a default plot when the data is selected using visualization tool.        

**Author Response**: We created default workspaces at https://epiviz.github.io/metaviz-workspaces/ with alpha diversity plots on either the disease or an interesting attribute.
